# Influence of inoculated gut bacteria on the development of *Bactrocera dorsalis* and on its susceptibility to the entomopathogenic fungus, *Metarhizium anisopliae*

**DOI:** 10.1186/s12866-020-02015-y

**Published:** 2020-10-21

**Authors:** Joseph Gichuhi, Fathiya Khamis, Johnnie Van den Berg, Samira Mohamed, Sunday Ekesi, Jeremy K. Herren

**Affiliations:** 1 International Centre of Insect Physiology and Ecology (icipe), Nairobi, Kenya; 2 Unit for Environmental Sciences and Management, North-West University, Potchefstroom, South Africa

**Keywords:** *Bactrocera dorsalis*, Metarhizium anisopliae, Gut bacteria, Lactococcus lactis

## Abstract

**Background:**

Symbiotic interactions between insects and bacteria have been associated with a vast variety of physiological, ecological and evolutionary consequences for the host. A wide range of bacterial communities have been found in association with the oriental fruit fly, *Bactrocera dorsalis* (Hendel) (Diptera: Tephritidae), an important pest of cultivated fruit in most regions of the world. We evaluated the diversity of gut bacteria in *B. dorsalis* specimens from several populations in Kenya and investigated the roles of individual bacterial isolates in the development of axenic (germ-free) *B. dorsalis* fly lines and their responses to the entomopathogenic fungus, *Metarhizium anisopliae*.

**Results:**

We sequenced 16S rRNA to evaluate microbiomes and coupled this with bacterial culturing. Bacterial isolates were mono-associated with axenic *B. dorsalis* embryos. The shortest embryonic development period was recorded in flies with an intact gut microbiome while the longest period was recorded in axenic fly lines. Similarly, larval development was shortest in flies with an intact gut microbiome, in addition to flies inoculated with *Providencia alcalifaciens.* Adult *B. dorsalis* flies emerging from embryos that had been mono-associated with a strain of *Lactococcus lactis* had decreased survival when challenged with a standard dosage of *M. anisopliae* ICIPE69 conidia. However, there were no differences in survival between the germ-free lines and flies with an intact microbiome.

**Conclusions:**

These findings will contribute to the selection of probiotics used in artificial diets for *B. dorsalis* rearing and the development of improved integrated pest management strategies based on entomopathogenic fungi.

**Supplementary information:**

**Supplementary information** accompanies this paper at 10.1186/s12866-020-02015-y.

## Background

The oriental fruit fly, *Bactrocera dorsalis* (Hendel) (Diptera: Tephritidae) is a pest of cultivated fruit and has been recorded in various locations across Asia, Africa, and North America and recently in Europe [1–9]. Since this species is considered a high risk quarantine pest, infestation with this pest has significant implications for production, trade and socio-economic aspects of affected countries [10–13].

The symbiotic association between insects and bacteria has important implications for insect physiology, ecology and evolution [14]. Therefore, understanding symbiont-host interactions in diverse groups of insects has become a priority. For major pest species, such as *B. dorsalis*, identification of associated bacterial communities can provide useful insights into biological characteristics and lead to improved control methods.

The ability of bacterial symbionts to influence the development of their host and to modulate the host’s response to entomopathogenic fungi has been demonstrated in a number of insect species. Endosymbionts as well as gut bacteria have been shown to affect the durations of embryonic and post-embryonic development periods [15–20]. Such associations have been linked to additional roles such as provision of essential amino acids and vitamins by bacteria to their insect hosts [18, 21]. In addition, symbiotic bacteria have been reported to exhibit inhibitory capabilities against fungal infections in *Drosophila melanogaster* [22, 23], ants [24], wasps [25] aphids [26–28] and beetles [29]. These capabilities could be effected via production of antifungal metabolites [30, 31] or other unknown mechanisms. For *B. dorsalis*, the entomopathogenic fungus, *Metarhizium anisopliae* (Metchnikoff) Sorokin is often applied as a biopesticide [32–36]. However, limited information is available regarding whether bacterial symbionts influence the response of *B. dorsalis* to this fungus.

A number of studies have investigated the diversity and structure of the gut microbiome of *B. dorsalis* [1, 37–45]. The specific roles of certain bacterial isolates from *B. dorsalis* have also been reported. For example, isolates have been shown to directly affect the nutrient ingestion and foraging behavior of *B. dorsalis* [39]*.* Some bacterial isolates, including *Enterococcus* sp., *Microbacterium*, *Klebsiella pneumoniae* and *Lactococcus lactis*, have been found to influence the developmental time, morphological parameters and survival of the oriental fruit fly [37]. Bacterial insolates have also been found to influence mate-selection behavior in *B. dorsalis* [46]*. Bactrocera dorsalis* also benefits from the ability of some of its gut associated bacteria to break down toxicants, which has been linked to insecticide resistance in this species [43, 47].

In this study, we isolated some common bacterial species associated with *B. dorsalis* populations in Kenya and investigated their roles in the development of immature stages of *B. dorsalis.* We also evaluated the implications of rearing flies supplemented with single bacterial isolates on the survival of adult flies when exposed to an entomopathogenic fungus.

## Results

The microbiomes of *B. dorsalis* specimens originating from different regions of Kenya were found to be dominated by the bacterial genera: *Lactobacillus, Klebsiella, Enterobacter, Providencia, Lactococcus* and *Pantoea* amongst several others (Supplementary Figs. 1 and 2). Three genera: *Enterobacter*, *Klebsiella* and *Serratia* (in adult specimens) and *Lactobacillus* (in larval specimens) were differentially abundant among the sampled locations (Supplementary Fig. 3). The abundance of the detected bacterial genera was found to differ among the sampled locations (Supplementary Fig. 4).

A total of 12 unique bacterial isolates were isolated through culturing of gut homogenates of adult and larval specimens from the five sampled sites: Embu, Muranga, Makueni, Kitui, Nguruman and the *icipe* laboratory colony. Similar to the 16S sequencing result, several isolates in the Enterobacteriaceae family (*Enterobacter cloacae*, *E. asburiae*, *E. tabaci*, *Klebsiella oxytoca, Providencia alcalifaciens* and *P. rettgeri*) were isolated mainly from sites in which high proportions of the respective genera had been detected. In addition, *L. lactis* strains were isolated from Embu and Nguruman specimens (Supplementary Table 1). Of these, *E. cloacae, K. oxytoca, L. lactis, P. alcalifaciens* as well as *Citrobacter freundii* were used to generate mono-associated fly lines.

**Fig. 1:**
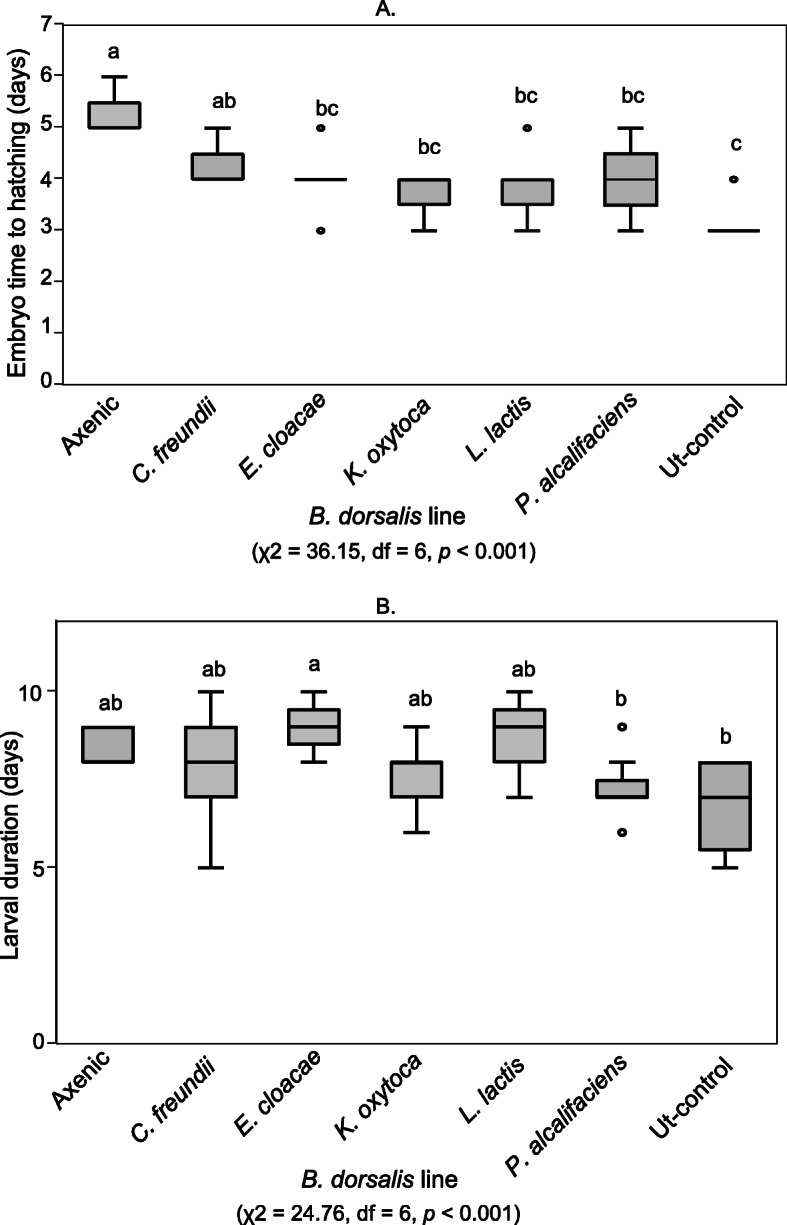
Boxplot of a Embryo hatching time and **b** Larval development duration of the *B. dorsalis* lines. Plots with the same letter are not significantly different (Dunn’s *p* > 0.05). The median is shown as a black line within the box. The edges of the box indicate the 25th and 75th percentiles. Whiskers span 1.5 times the interquartile range. Outliers of individual variables are represented as circles. Untransformed data are shown

We evaluated developmental and fitness measures such as pupal size and weight in *B. dorsalis* lines inoculated with the respective bacterial isolates. Significant variations in the time taken for embryos to hatch were recorded between the different *B. dorsalis* lines (χ2 = 36.15, df = 6, *p* < 0.001). Embryos of the *B. dorsalis* lines with an intact microbiome (hereafter referred to as Ut-control) were observed to take the shortest duration to hatch whereas the longest duration was recorded in the axenic line (Fig. 1a). Similarly, the *B. dorsalis* lines had significantly different durations at larval stage (χ2 = 24.76, df = 6, *p* < 0.001), with the Ut-control and the *P. alcalifaciens* line having the shortest duration*.* (Fig. 1b).

**Fig. 2:**
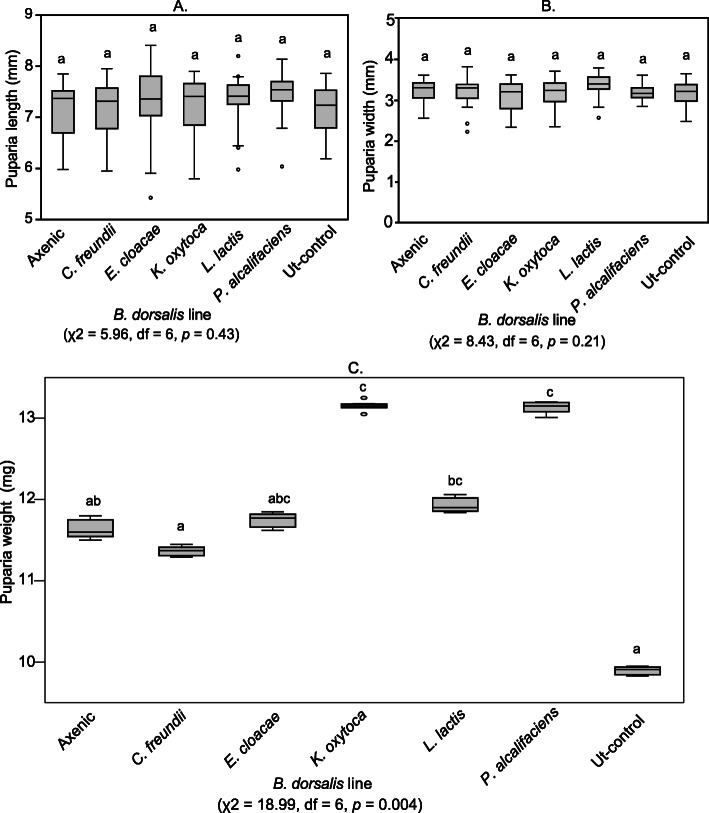
Boxplots of puparia (**a**) lengths (**b**) widths and (**c**) weights of the *B. dorsalis* lines. Plots with the same letter are not significantly different (Dunn’s *p >* 0.05). The median is shown as a black line within the box. The edges of the box indicate the 25th and 75th percentiles. Whiskers span 1.5 times the interquartile range. Outliers of individual variables are represented as circles. Untransformed data are shown

We examined the puparia size of inoculated *B. dorsalis* lines as a proxy for assessing the effects of microbiota members on the host fitness. There was no significant difference in means of puparia lengths among all the fly lines (χ2 = 5.96, df = 6, *p* = 0.43) (Fig. 2a). Similarly, none of the fly lines exhibited a significant variation in width of puparia (χ2 = 8.43, df = 6, *p* = 0.21) (Fig. 2b). However, significantly different puparia weights were recorded among the *B. dorsalis* lines (χ2 = 18.99, df = 6, *p* = 0.004) except between the *K. oxytoca* and the *P. alcalifaciens* lines as well as between the Ut-control and the *C. freundii* line (Fig. 2c).

**Fig. 3:**
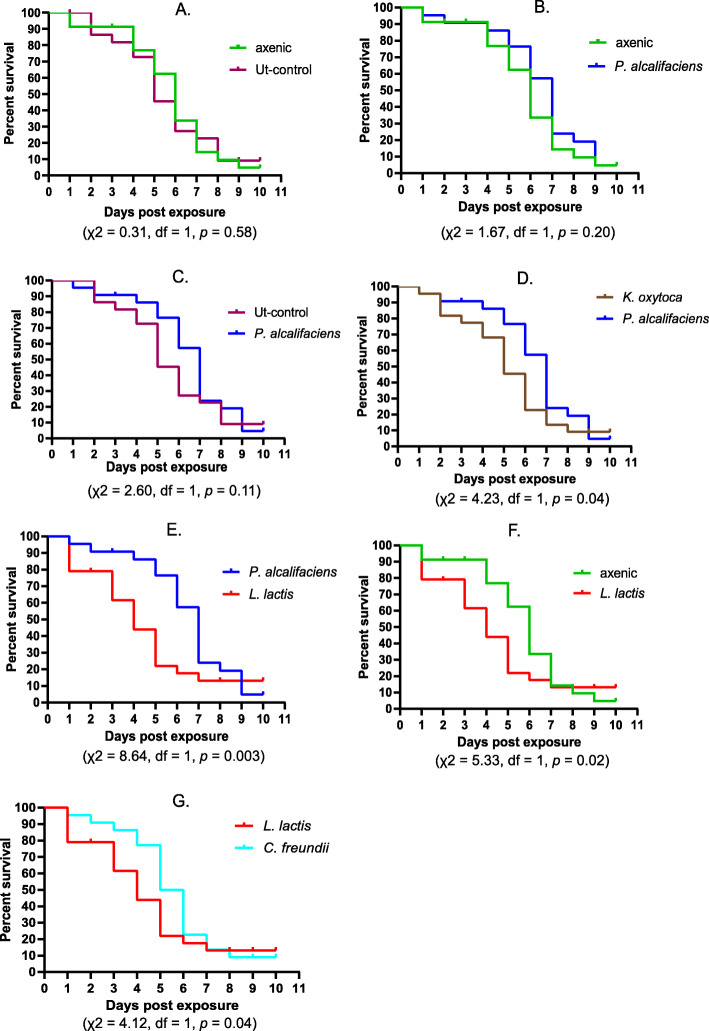
Survival comparison between inoculated *B. dorsalis* lines, post exposure to *M. anisopliae* as adult flies. **a** Axenic and Ut-control **b** *P. alcalifaciens* line and axenic control **c** *P. alcalifaciens* line and Ut-control **d** *P. alcalifaciens* and *K. oxytoca lines* **e** *P. alcalifaciens* and *L. lactis* lines **f** *L. lactis* line and axenic control, and **g** *L. lactis* and *C. freundii* lines

In addition, we investigated the survival rates of adult flies emerging from the bacterial inoculated *B. dorsalis* lines and controls, upon infection with the entomopathogenic fungus, *Metarhizium anisopliae*, ICIPE69*.* No significant variations in survival were recorded between the axenic control and the Ut-control (χ^2^ = 0.31, df = 1, *p* = 0.58). However, survival of the axenic line varied significantly from the *L. lactis* line (χ2 = 5.33, df = 1, *p* = 0.02). Although survival of the *P. alcalifaciens* line did not vary significantly from that of the axenic control (χ2 = 1.67, df = 1, *p* = 0.20) and the Ut-control (χ2 = 2.60, df = 1, *p* = 0.11), significant variation in survival was recorded between this line and the *K. oxytoca* line (χ2 = 4.23, df = 1, *p* = 0.04) as well as with the *L. lactis* line (χ2 = 8.64, df = 1, *p* = 0.003). In addition to the axenic control as aforementioned, the *L. lactis* line also varied significantly in survival from the *C. freundii* line (χ2 = 4.12, df = 1, *p* = 0.04) as shown in Fig. 3. However, survival between the rest of the *B. dorsalis* lines and controls did not vary significantly (Supplementary Table 2).

**Fig. 4:**
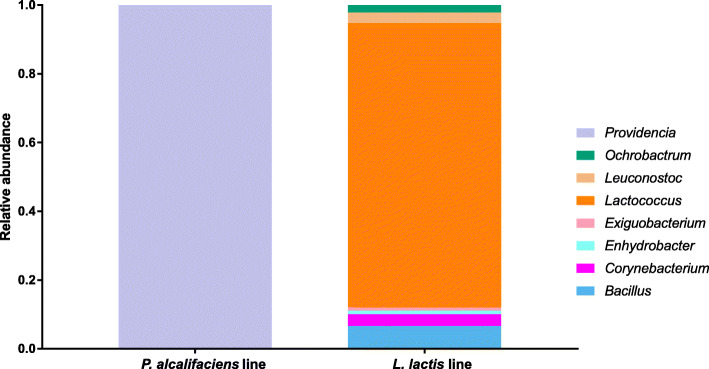
Relative abundances of bacterial genera in the gut tissues of adult flies from the *B. dorsalis* lines inoculated with P*. alcalifaciens* and *L. lactis* respectively

The *B. dorsalis* line inoculated with *L. lactis* exhibited a significant diminished survival at adult stage (Fig. 3f) whereas the line inoculated with *P. alcalifaciens* showed a slightly improved survival from days 4 to 7 (Fig. 3b and c) which, however, did not significantly affect the overall survival of this line. We therefore investigated the structure of the gut microbiota of adult flies emerging from these two lines, unexposed to the fungus. The microbiome of 2-day old adult flies from the *L. lactis* fly line was found to consist mainly of *Lactococcus* and lesser proportions of other bacterial genera, whereas that of adults from the *P. alcalifaciens* line was fully colonized by *Providencia* (Fig. 4)*.* Notably, the observed microbiome compositions at adult stages of the tested lines are highly reflective of the bacterial isolates that were introduced during the immature stages. In the *L. lactis* mono-associated fly line, we observed a low proportion of other bacterial groups suggesting that very limited re-colonization from other environmental bacteria occurred.

## Discussion

The gut microbiome of *B. dorsalis* derived from different populations in Kenya was found to consist of members that have been reported among the gut microbiota of *B. dorsalis* in other parts of the world [1, 22–31]. We found that *B. dorsalis* can be re-inoculated with bacterial isolates at the embryo stage and that the effects of such isolates on host development and susceptibility to pathogens can subsequently be evaluated.

Broad-spectrum antibiotics are often employed in studies to disrupt the gut microbiota of insects. In addition, antibiotics may have detrimental effects on host organisms, for instance the interference of the protein synthesis mechanisms of the host [48]. The dechorionation of embryos coupled with rearing on germ-free diets has been reported to be more effective in eliminating bacteria from insect eggs than the use of antibiotics [48]. For this reason, we adopted a dechorionation approach to generate axenic flies for mono-association experiments.

The absence of bacteria in the axenic lines was observed to significantly lengthen the period taken for embryos to hatch compared to *B. dorsalis* lines inoculated with individual bacterial isolates, as well as the Ut-control which had an intact microbiome. This suggests that the effect of bacterial isolates on hatching time may be combinatorial, or possibly there are isolates that have stronger effects but these were not isolated in our experiments. However, the mechanisms through which embryonic development and bacteria intersect are still unclear. In other insects, microbiomes have been associated with hatching duration for both host and parasite eggs [49, 50]*.*

The shortest larval development period was recorded in flies with an intact microbiome as well as in the *P.* *alcalifaciens* line whereas the longest period was recorded in the *E. cloacae* fly line. In other insects, *E. cloacae* has been shown to stimulate the host’s immune responses which can protect the host from other pathogens or in some others it causes pathogenesis and mortality [51–53]. In *B. dorsalis*, this bacterium has been reported to increase fecundity but lower the longevity of adult flies [39]. Since this bacterium has frequently been detected as a member of the *B. dorsalis* gut microbiota, it may be that the host benefits from accommodating this bacterium, despite the decreased longevity because of its enhancement of reproductive capacity. In comparison, *L. lactis* has also been reported to prolong larval development of *B. dorsalis* when supplemented in diet of non-axenic flies [37]. In this study, *L. lactis* inoculated in *B. dorsalis* did not cause significant variation in duration of the larval stage relative to other isolates and control conditions.

The observed variations in development time of various stages of an insect host could be directly influenced by some dominant roles played by bacterial symbionts in the host’s physiology, immune homeostasis, detoxification, and in nutrient provisioning and utilization. Of these, proteomics evidence suggests that the most dominant role played by bacteria is amino acid synthesis, followed by protein digestion, energy metabolism, vitamin biosynthesis, lipid digestion, plant secondary metabolite degradation, and carbohydrate digestion [54]. Future studies could evaluate the intricate mechanisms of bacterial influence in the development of important pests such as *B. dorsalis*.

Minor but significant effects of bacterial isolates on puparia weight were recorded, however, no significant differences in puparia length and width measurements were recorded amongst all the inoculated *B. dorsalis* lines. It could be possible that these are not useful parameters for testing fitness variations in *B. dorsalis.* The lower puparia weight recorded in the Ut-control relative to majority of *B. dorsalis* lines with re-introduced bacteria supports a previous report which demonstrated that supplementing larval diets with certain gut bacteria results in a significant increase in puparia weight for *B. dorsalis* [37] as well as for the Mediterranean fruit fly, *Ceratitis capitata* (Wiedemann) (Diptera: Tephritidae) [55]. Increased pupal weight due to addition of probiotics has been associated with increased adult size and mating success of males [55]. Inclusion of *K. oxytoca* and *P. alcalifaciens* which promoted the highest pupal weight in this study as probiotics in rearing diets might therefore be useful in sterile insect technique (SIT) programs that capitalize on male mating success, since the high pupal weight gain in this study was indiscriminate of sex of the flies. Furthermore, it has been demonstrated that *K. oxytoca* can repair the ecological fitness damage caused by irradiation of *B. dorsalis*, as well as improve food intake and elevate sugar and amino acid levels in the haemolymph of irradiated flies [45].

We recorded significant differences in survival of *B. dorsalis* flies after exposure to the entomopathogenic fungus, *M. anisopliae.* Notably, the *B. dorsalis* line inoculated with *L. lactis* exhibited poor survival relative to the axenic control and two other isolates. The *L. lactis* could therefore have some level of *M. anisopliae*-synergistic pathogenicity to *B. dorsalis* at adult stage resulting in poor survival of exposed flies. In other studies, *L. lactis* has been demonstrated to have pathogenic effects when supplemented in larval rearing diets of *B. dorsalis*, resulting in overall decreased survival of flies [37]*.* However, this effect might be strain specific, since no overt pathogenic effects such as decreased survival rates were recorded in the presence of *L. lactis* without *M. anisopliae* in our experiments. Under natural conditions, it is possible that *L. lactis* strains cause low but variable levels of pathogenicity to adult *B. dorsalis.* Also, *B. dorsalis* could have adaptive decoupling where immature stages mount a stronger response against *L. lactis* than adult stages. Such has been demonstrated in mosquitoes [56]. In addition, *L. lactis* has been described as a non-obligate pathogen capable of achieving high bacterial loads during infection yet result in low mortality in a *Drosophila* model [57]. This could relate to our finding that *Lactococcus* was dominant in wild flies from Embu and Nguruman, as well as with other studies that have detected this bacterium in *B. dorsalis* [1, 37, 38, 42, 43, 45, 58]. This indicates that *L. lactis* is ordinarily accommodated as a member of the *B. dorsalis* microbiota due to possible benefit to larvae (or adults) which balances out a fitness cost to adults. Alternatively, it is possible that *L. lactis* is a low virulence pathogen that is overall detrimental to *B. dorsalis* under natural conditions.

Transstadial persistence of various gut bacteria has been demonstrated in *B. dorsalis* [44, 59, 60]. Following the significant reduced survival and the relative improved survival between day 4 and 7 of adult flies exposed to *M. anisopliae* for the *L. lactis* and *P. alcalifaciens* inoculated *B. dorsalis* lines respectively, we evaluated the persistence of the re-introduced bacteria, at the adult stage. The re-introduced bacteria in axenic flies were found to persist from immature stages to adult stages of both the *L. lactis* and *P. alcalifaciens* inoculated *B. dorsalis* lines, with the latter line having a complete domination of gut tissues by the inoculated bacteria. Since immature stages of both *B. dorsalis* lines were reared in similar axenic conditions whereas adult flies were maintained under normal conditions, the presence of lesser quantities of other bacteria in the *L. lactis* line and not in the *P. alcalifaciens* line indicate a stronger competitive inhibitory effect of the latter isolate to proliferation of environmental microbes in the 2-day foraging window between eclosion and testing. The effects of supplementation of larval diets with such gut bacteria are therefore likely to persist across generations and populations since the transstadially transmitted bacteria could also be transmitted horizontally and through egg surface contamination as reported for some gut symbionts in *B. dorsalis* [61, 62].

## Conclusion

This study demonstrates the role of gut bacteria in the development of immature stages of *B. dorsalis* as well as a synergistic effect between the gut bacterium, *L. lactis* and a commonly used biopesticide, *M. anisopliae,* in decreasing the survival of adult stages of this pest. These findings reveal some profound effects of certain bacterial isolates on the biology of *B. dorsalis.* This can inform probiotic selection and development for *B. dorsalis* rearing diets and also be applied in integrated pest management programs to increase the efficiency of entomopathogenic fungi.

## Methods

### 16S rRNA sequencing

To assess the broader composition of gut microbiota associated with *B. dorsalis* populations in five mango growing regions in Kenya, high throughput sequencing of the bacterial 16S rRNA gene was carried out for adult samples collected in 2016 and third stage larval samples collected in 2018, retrieved from Kent variety mangoes, with inclusion of a laboratory reared (*icipe*) colony for comparison. Infested mangoes were collected from farms in Embu (S 0° 28′ 56.6“ E 37° 34’ 55.5”), Muranga (S 0° 42′ 50.0“ E 37° 07’ 03.4”), Nguruman (S 1° 48′ 32″ E 36° 03′ 35″), Makueni (S 2° 21′ 18.9576″ E 38° 11′ 26.376″) and Kitui (S 01° 21′ E 38° 00′).

At each sampling, infested mangoes were washed in distilled water, dissected and placed on sterile sand in ventilated cages at 27 ± 2 °C and 70% humidity to allow third stage larvae to burrow and pupate in sand. Puparia were retrieved from sand through sieving and maintained in sterile petri dishes in ventilated Perspex cages until eclosion. A proportion of third instar larvae were directly retrieved from the fruit for gut dissection. Emerging 1 day old adult flies from respective sites were collected for gut dissections.

Guts were dissected in sterile phosphate buffered saline (PBS) (140 mM NaCl, 2.7 mM KCl, 10 mM Na_2_HPO_4_·7H_2_O, and 1.8 mM KH_2_PO_4_ [pH 7.4]) after surface sterilization of the specimens. The selected larvae and adult flies were surface sterilized as described previously [63]. Dissected guts were homogenized using pestles in 1 ml microfuge tubes containing 300 μl PBS.

A total of five adult specimens per site sampled in 2016, and four larval specimens per site sampled in 2018 were randomly selected for DNA extraction. In addition, five adult flies and four larval specimens were included from the International Centre of Insect Physiology and Ecology (*icipe*) fruit fly laboratory colony. The sampled colony was derived from infested mango collected from different farms across Kenya and maintained for more than 40 generations with frequent wild infusions in the laboratory at 27 °C and 60% relative humidity. Adult flies were fed on a diet consisting of 3 parts sugar and 1-part enzymatic yeast hydrolysate ultrapure (USB Corporation, Cleveland, Ohio, USA), and water on pumice granules. For each generation, fresh mango domes were used as oviposition receptacles, from which embryos were washed in distilled water before inoculation on larval rearing diets [64]. Same age and generation of laboratory reared flies were used for this study.

DNA extraction and high throughput sequencing were carried out as previously described [63]. Larval DNA was sequenced at the Centre for Integrated Genomics, University of Lausanne, Switzerland and adult DNA at the Macrogen Europe Laboratory, the Netherlands. Adult and larval sequence sets were therefore analyzed separately.

### 16S rRNA gene sequence analysis

Sequence reads were quality checked and pre-processed in QIIME2 [65] as described previously [63]. A total of 638,815 sequence reads from adult specimens and 56,425 from larval specimens that were retained after removal of spurious reads and all reads shorter than 240 and 272 nucleotides in length respectively, were subjected to further analysis. These sequences clustered into 235 OTUs (adult) and 402 OTUs (larval). Of these, 50 OTUs (adult) and 94 OTUs (larval) survived low count and interquartile range-based variance filtering to eliminate OTUs that could arise from sequencing errors and contamination. Taxonomic assignment, OTU variance filtering and beta diversity measures were carried out as previously described [63]. Differential abundance of bacterial genera was evaluated using the differential gene expression analysis based on the negative binomial distribution (DESeq 2) tool [66].

### Bacterial isolation

Cultivable bacteria were isolated from gut homogenates of both larvae and adult flies collected during 2018 from the aforementioned sites in Kenya. Larvae and adults were retrieved from infested mangoes and their guts dissected and homogenized as described above. An aliquot of 5 μl of the fourth serial dilution of each homogenate was inoculated under aerobic conditions on brain heart infusion (BHI) solid media using the spread plate technique [67] and incubated at 37 °C for 14 h. Representative colony forming units (cfu) were selected based on morphology and clonally propagated up to four times to ensure purity on BHI agar plates.

### Bacterial isolates identification

Pure isolates were sub-cultured in BHI broth and incubated at 37 °C for 16 h on a shaking platform at 300 rpm. Bacterial cells were harvested from media then washed thrice in PBS by centrifugation at 10000 rpm for 10 min at 10 °C, each time discarding the supernatant.

DNA extraction from bacterial cells and PCR amplification were carried out as described previously [63] with slight variations in the primers used i.e. the 28F (5′-GAGTTTGATCNTGGCTCAG-3′) and 519R (5′-GTNTTACNGCGGCKGCTG-3′) primer pair, as well in the cycling conditions, where, following the initial denaturation, 35 cycles of 30 s at 95 °C, 40 s at 54 °C and 1 min at 72 °C were run, followed by the final elongation step. Direct Sanger sequencing in both forward and reverse directions was done for all amplified samples. Sequence alignments were performed using Clustal W in Geneious 8.1.9 software [68]. Homology searches using BLAST against the 16S ribosomal RNA sequence database at the National Center for Biotechnology Information (NCBI) were done to infer identity and similarity of isolates to subject sequences in the database.

### Generation of axenic lines

*Bactrocera dorsalis* embryos were collected from gravid females from the *icipe B. dorsalis* laboratory reared colony using perforated mango domes. Embryos were surface sterilized in 70% ethanol for 5 min, then dechorionated in a 7% v/v sodium hypochlorite solution for 3 min in a fine mesh (Nitex Nylon100 μm) basket. Dechorionated embryos were rinsed three times in distilled water for 5 min each then flooded with absolute ethanol. Sterile materials were used in subsequent procedures in a sterile laminar flow hood. Using a fine camel hair brush, embryos from the bottom of the basket were transferred and spread out on 2 cm X 2 cm X 4 mm sponge cloth immersed in larval rearing diet [64] in flat base 30 mm X 100 mm cylindrical test tubes. Approximately 100 embryos were placed in each tube. Axenic control lines were derived at this step by plugging cotton wool up to 3 cm from the top of the tube.

### Generation of mono-association lines

An inoculum 50 μl of 1 X 10^4^ cfu/ml of each isolate was introduced in triplicate per experiment directly onto the embryos before plugging the tubes with cotton wool.

### Rearing and quality check of fly lines

All tubes were maintained at 27 °C and 70% humidity. A control group with an intact microbiome (whose embryos were not dechorionated) was included in triplicate in each experiment. The immature stages of all *B. dorsalis* lines were reared in axenic conditions.

To quality check axenic lines, random third stage larvae were retrieved from axenic control tubes per experiment and homogenized in 50 μl PBS. Five μl of this homogenate was plated on nutrient agar plates and incubated at 37 °C and checked for bacterial growth after 15 h. A volume of 200 μl of sterile larval rearing diet was added to each tube every 24 h after hatching.

No bacterial growth from third instar larvae was recorded on enriched media plates during quality check of axenic lines, inferring a strong elimination effect using this approach.

Mature third-stage larvae crawled upward and burrowed into the cotton wool plug to pupate. The time taken to the observation of at least 10 first instar larvae as well as to the observation of puparia on the cotton wool plug were recorded for each tube. Measurements of 1-day old puparia weight, dorsal to ventral length as well as width of the sixth segment were also recorded. Weights were recorded in triplicates of 20 puparia each from every fly line, whereas length and width measurements were recorded from 20 puparia per fly line. Length measurements were carried out under a Leica LAS EZ4D stereomicroscope (Leica Ltd., Switzerland). Cotton wool plugs with puparia were submerged in autoclaved distilled water at room temperature and carefully pulled apart to free puparia. The retrieved puparia were dried on sterile paper towel and maintained on sterile Petri dishes placed in sterilized ventilated Perspex cages until eclosion at 27 °C and 70% humidity. Eclosed adult flies were maintained under normal conditions. All data for development and puparia measurements were tested for normality using the Shapiro-Wilk’s test. Non-normal distributions were recorded in the embryo duration (W = 0.84, *p* < 0.001), larval duration (W = 0.92, *p* < 0.001), puparia length (W = 0.93, *p* < 0.001), puparia width (W = 0.96, *p* < 0.001) and puparia weight (W = 0.87, *p* < 0.001) datasets. All datasets conformed to homogeneity of variance as determined using the Levene’s test: embryo duration (F_(6, 56)_ = 0.50, *p* = 0.81), larval duration (F_(6, 56)_ = 1.24, *p* = 0.30), puparia length (F_(6, 133)_ = 0.85, *p* = 0.54), puparia width (F_(6, 133)_ = 1.31, *p* = 0.26) and puparia weight (F_(6, 56)_ = 1.34, *p* = 0.25). Statistical significance in the datasets was therefore evaluated using the Kruskal-Wallis test followed by Dunn’s multiple comparisons post hoc test. All analyses were conducted in the R statistical software [69]. In addition, adult flies emerging from all mono associated lines were monitored for fitness for a period of 12 days post eclosion. No mortality was recorded in any of the *B. dorsalis* lines during this period.

### Exposure to Metarhizium anisopliae

Dry conidia of *M. anisopliae* ICIPE69 were obtained from *icipe*’s Arthropod Pathology Unit Germplasm. Triplicates in groups of 20 newly emerged adult flies from each fly line were each exposed to 0.3 g of dry spores of the *M. anisopliae* ICIPE69 for 1 min in a contamination device made from a 50 ml falcon tube lined with velvet. Exposed flies were released in 10 cm × 10 cm × 10 cm ventilated cages and maintained on adult *B. dorsalis* rearing diet [70] and sterile water saturated on cotton wool at 27 °C and 70% humidity. A control set derived from unexposed flies was included in each treatment. All flies in this set (unexposed to fungus) in all *B. dorsalis* lines survived the duration of the experiment. The survival rates of *B. dorsalis* from the different fly lines were monitored daily after exposure to the *M. anisopliae* isolate. Pairwise comparisons of survival between all the fly lines and controls was evaluated using the Gehan-Breslow-Wilcoxon test. Survival curves for adult flies exposed to ICIPE69 were generated using the Kaplan-Meier method in the Graph Pad Prism software, version 7.00 for Windows [71].

### Fly line microbiota

Gut tissues from a pool of five 2-day post eclosion adult flies per line from the *L. lactis* and the *P. alcalifaciens* inoculated *B. dorsalis* lines were processed as described above for high throughput sequencing, targeting the v3-v4 region of the bacterial 16S rRNA gene, and similarly analyzed in QIIME2–2018.11.

## Supplementary information

**Additional file 1 Supplementary Fig. 1.** Relative abundance of bacterial genera in adult *B. dorsalis* specimens sampled from different sites in Kenya.

**Additional file 2 Supplementary Fig. 2.** Relative abundance of bacterial genera in larvae specimens of *B. dorsalis* collected from different sites in Kenya.

**Additional file 3 Supplementary Fig. 3.** Differential abundance of A) *Enterobacter* B) *Klebsiella* C) *Serratia* in adult specimens, and D) *Lactobacillus* in larvae of *B. dorsalis* sampled from different sites in Kenya.

**Additional file 4 Supplementary Fig. 4.** Principal coordinate analysis (PCoA) ordination based on Bray-Curtis dissimilarity matrices (A and C) and Unweighted UniFrac distance matrices (B and D) showing significantly different microbial compositions among different sites. Plots A and B show compositions in adult specimens while C and D show compositions in larval specimens of *B. dorsalis*. Significance values are indicated in each plot. Individual specimens are represented as dots colored according to sampling site.

**Additional file 5 Supplementary Table 1.** Description of identified bacterial isolates and their GenBank accession numbers.

**Additional file 6 Supplementary Table 2:** Pairwise comparison of survival rates between bacterial-inoculated *B. dorsalis* lines, post exposure to entomopathogenic fungus.

## Data Availability

The datasets generated and analyzed during the current study are available in the Sequence Read Archive (SRA) under the BioProject: PRJNA545161 and in the GenBank, accessions MK968291-MK968302.

## Abbreviations

PBS: Phosphate buffered saline
OTU: Operational taxonomic unit
BHI: Brain heart infusion
cfu: Colony forming unit
rpm: Revolutions per minute
SIT: Sterile insect technique
*icipe*: International Centre of Insect Physiology and Ecology
