# Supplementary figures and images for: Influence of inoculated gut bacteria on the development of Bactrocera dorsalis and on its susceptibility to the entomopathogenic fungus, Metarhizium anisopliae
Source: BMC Microbiol. 2020 Oct 21;20:321. doi: 10.1186/s12866-020-02015-y (PMC7579797; doi:10.1186/s12866-020-02015-y)

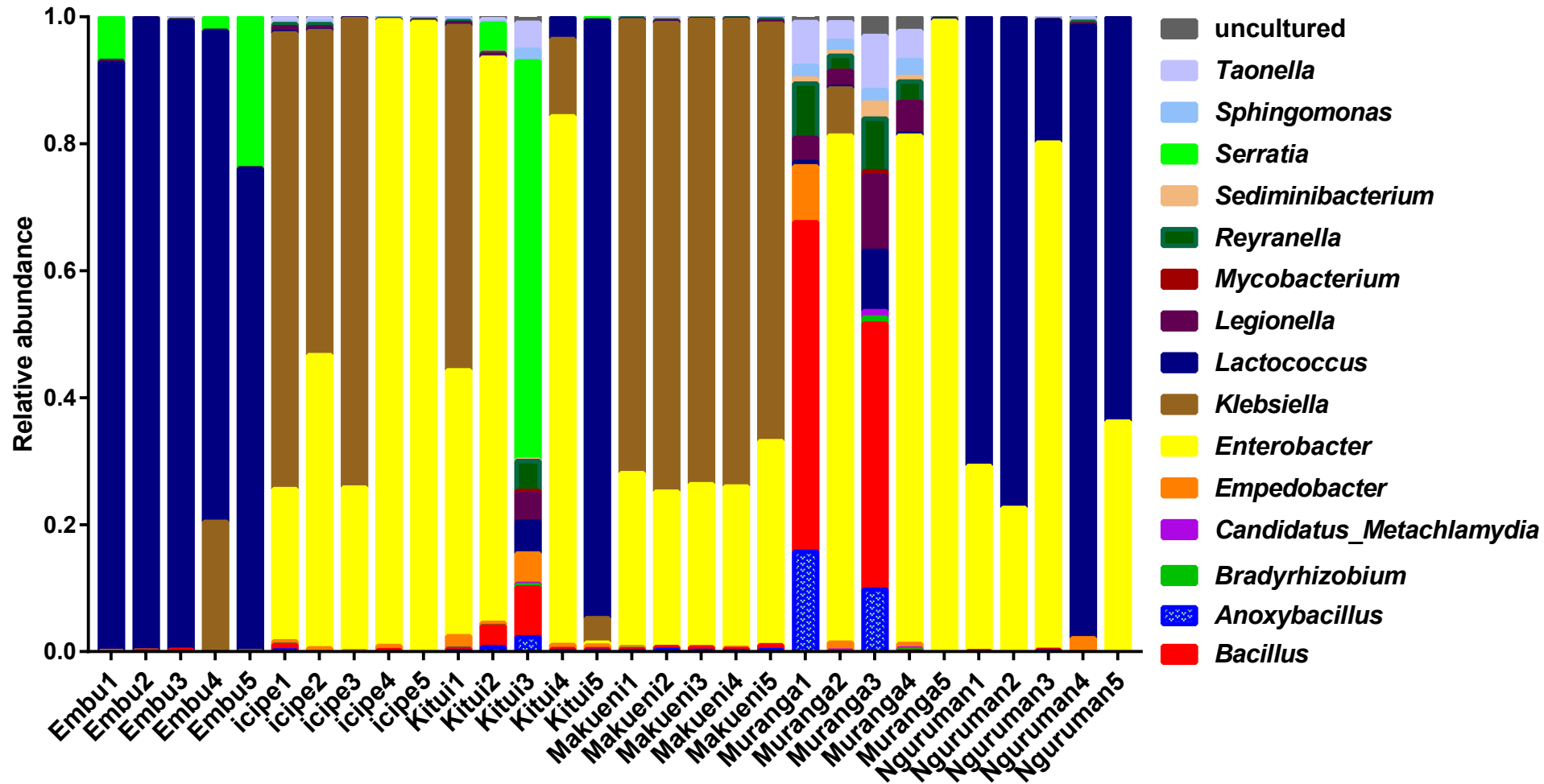

Supplement: Supplementary file 1 — Additional file 1 Supplementary Fig. 1. Relative abundance of bacterial genera in adult B. dorsalis specimens sampled from different sites in Kenya. [file 12866_2020_2015_MOESM1_ESM.pdf]

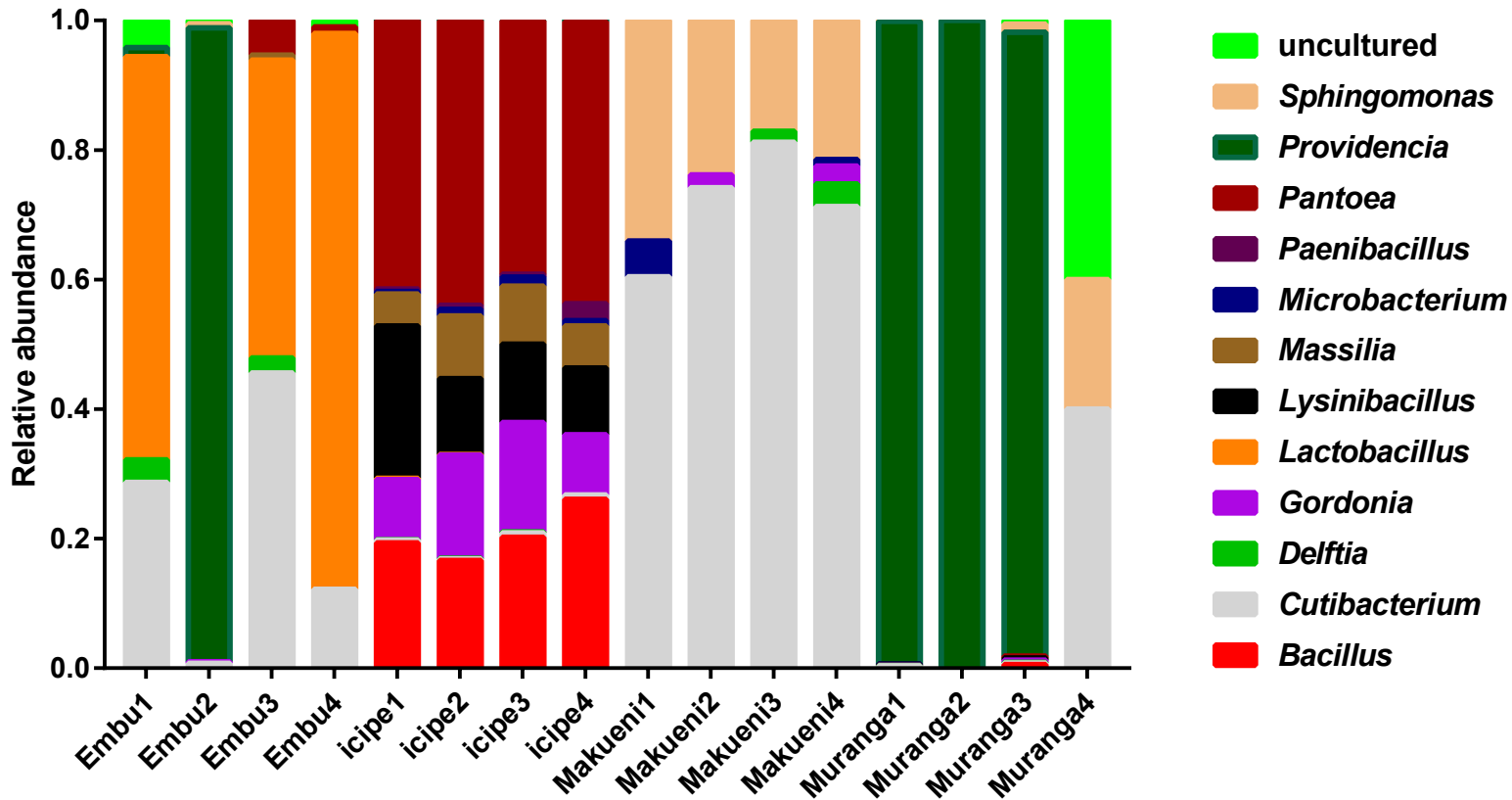

Supplement: Supplementary file 2 — Additional file 2 Supplementary Fig. 2. Relative abundance of bacterial genera in larvae specimens of B. dorsalis collected from different sites in Kenya. [file 12866_2020_2015_MOESM2_ESM.pdf]

A.

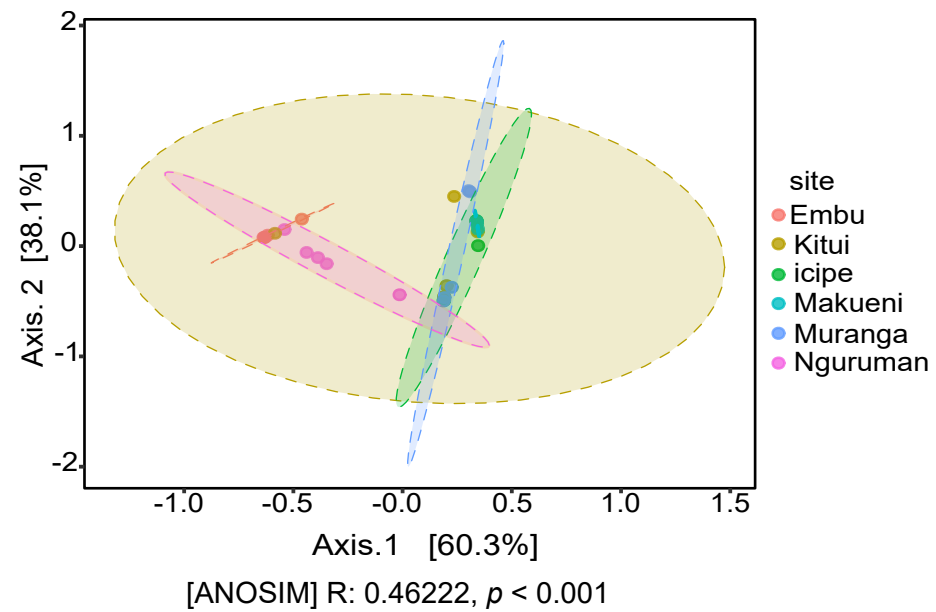

B.

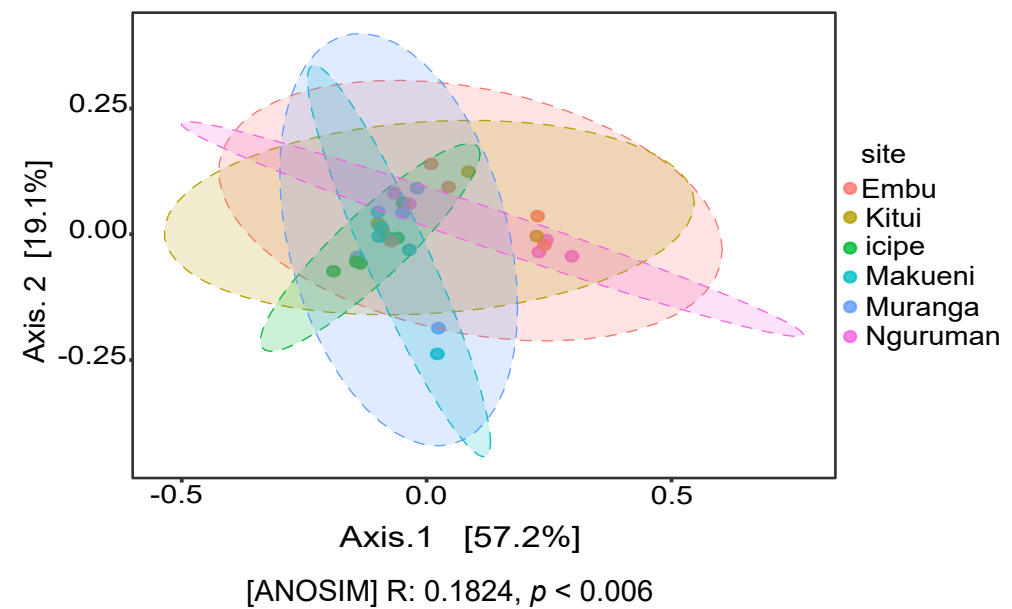

C.

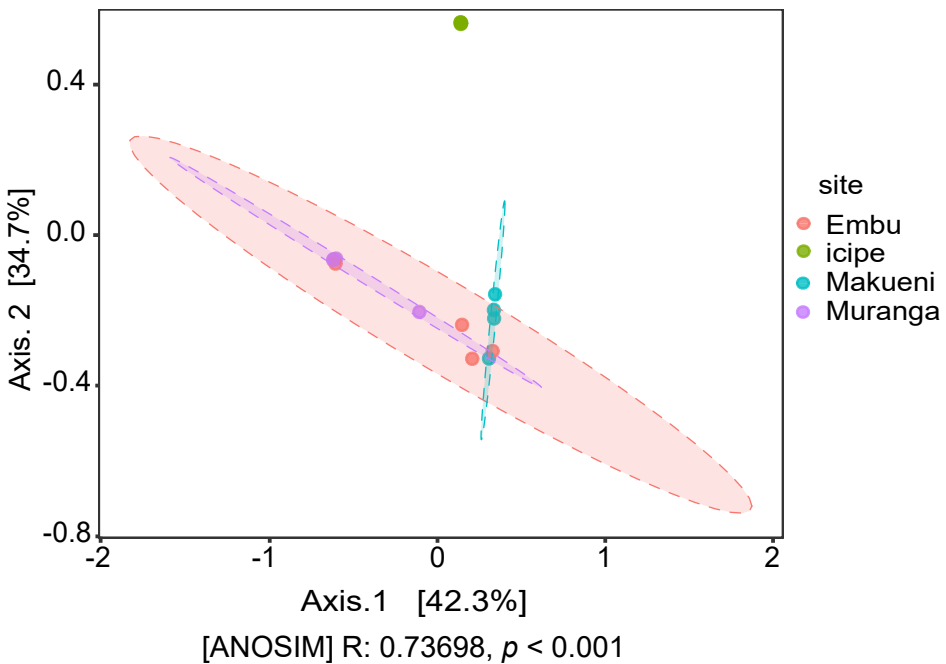

D.

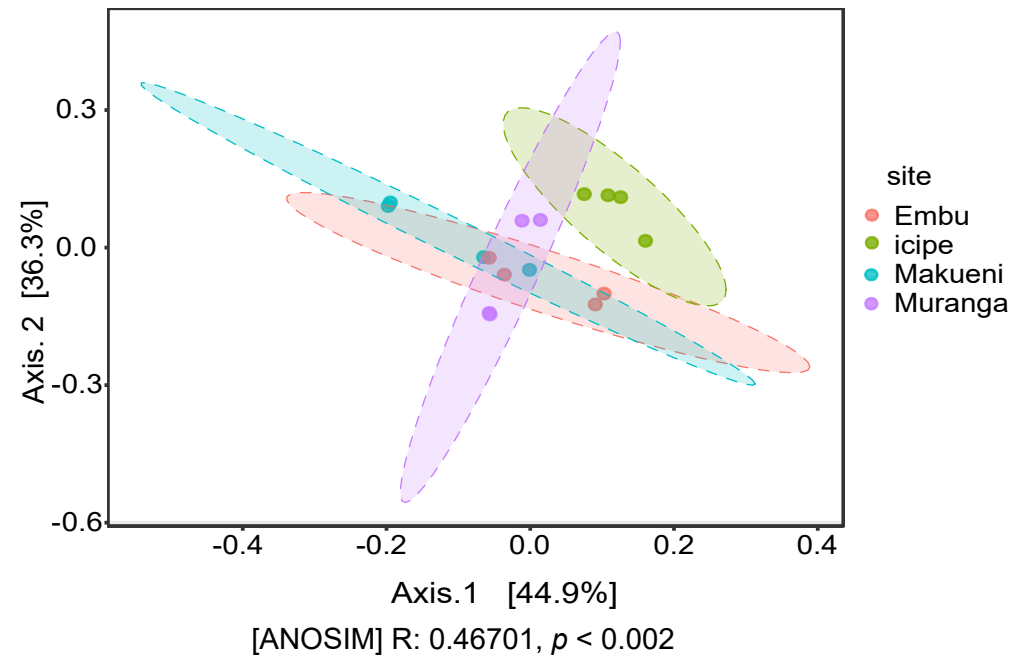

Supplement: Supplementary file 4 — Additional file 4 Supplementary Fig. 4. Principal coordinate analysis (PCoA) ordination based on Bray-Curtis dissimilarity matrices (A and C) and Unweighted UniFrac distance matrices (B and D) showing significantly different microbial compositions among different sites. Plots A and B show compositions in adult specimens while C and D show compositions in larval specimens of B. dorsalis. Significance values are indicated in each plot. Individual specimens are represented as dots colored according to sampling site. [file 12866_2020_2015_MOESM4_ESM.pdf]
