# Supplementary material for: Influence of inoculated gut bacteria on the development of Bactrocera dorsalis and on its susceptibility to the entomopathogenic fungus, Metarhizium anisopliae
Source: BMC Microbiol. 2020 Oct 21;20:321. doi: 10.1186/s12866-020-02015-y (PMC7579797; doi:10.1186/s12866-020-02015-y)
Supplement: Supplementary file 3 — Additional file 3 Supplementary Fig. 3. Differential abundance of A) Enterobacter B) Klebsiella C) Serratia in adult specimens, and D) Lactobacillus in larvae of B. dorsalis sampled from different sites in Kenya. [file 12866_2020_2015_MOESM3_ESM.pdf]

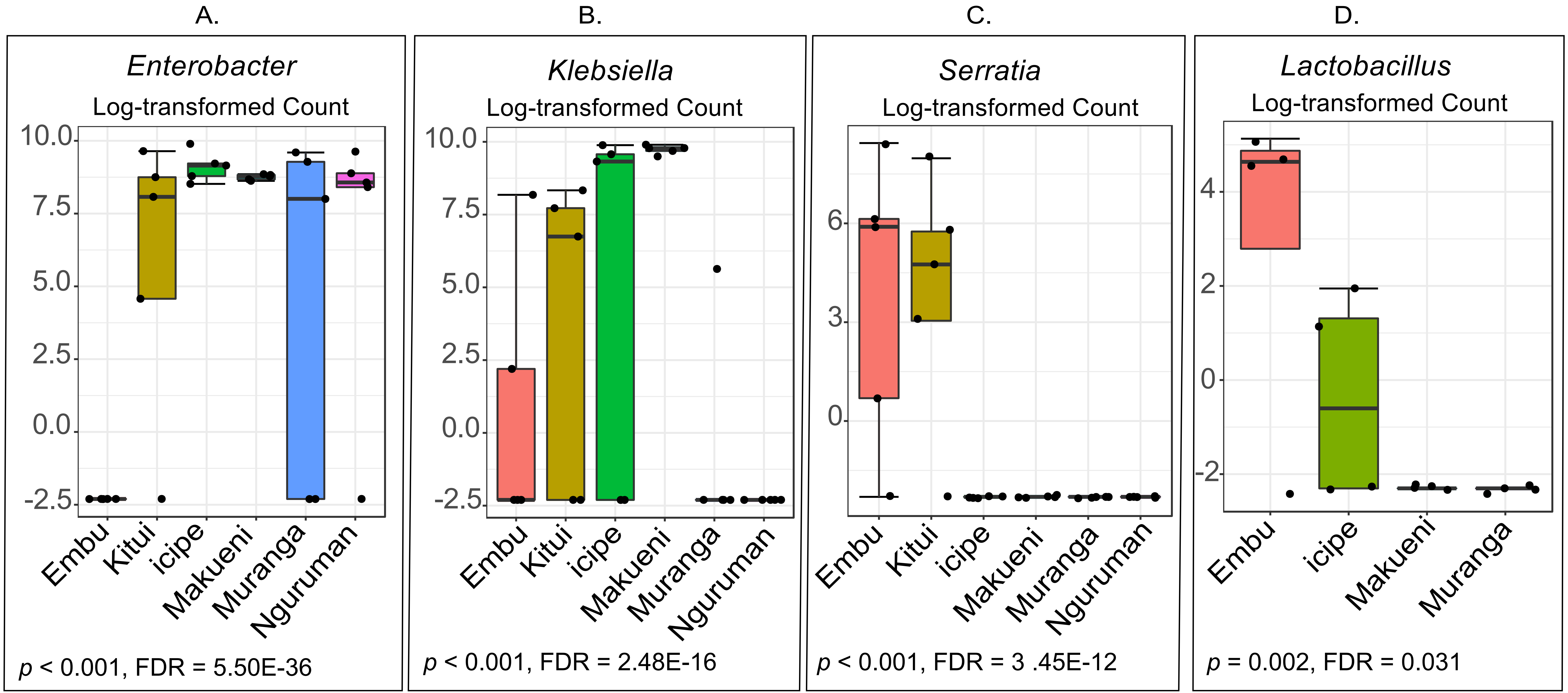
A.
Enterobacter
Log-transformed Count
10.0
7.5
5.0
2.5
0.0
-2.5
Embu
Kitui
icipe
Makueni
Muranga
Nguruman
p < 0.001, FDR = 5.50E-36
B.
Klebsiella
Log-transformed Count
10.0
7.5
5.0
2.5
0.0
-2.5
Embu
Kitui
icipe
Makueni
Muranga
Nguruman
p < 0.001, FDR = 2.48E-16
C.
Serratia
Log-transformed Count
6
3
0
Embu
Kitui
icipe
Makueni
Muranga
Nguruman
p < 0.001, FDR = 3 .45E-12
D.
Lactobacillus
Log-transformed Count
4
2
0
-2
Embu
icipe
Makueni
Muranga
p = 0.002, FDR = 0.031
