# Supplementary material for: Influence of inoculated gut bacteria on the development of Bactrocera dorsalis and on its susceptibility to the entomopathogenic fungus, Metarhizium anisopliae
Source: BMC Microbiol. 2020 Oct 21;20:321. doi: 10.1186/s12866-020-02015-y (PMC7579797; doi:10.1186/s12866-020-02015-y)
Supplement: Supplementary file 5 — Additional file 5 Supplementary Table 1. Description of identified bacterial isolates and their GenBank accession numbers. [file 12866_2020_2015_MOESM5_ESM.docx]

Supplementary Table1. Description of identified bacterial isolates and their GenBank accession numbers. (* utilized in mono-association lines)

| **Isolate** | **Accession** | **Phylum** | **Family** | **Closest match** | **% Identity** | **Source (location/specimen stage)** |
| --- | --- | --- | --- | --- | --- | --- |
| BD1* | MK968291 | Proteobacteria | Enterobacteriaceae | *Citrobacter freundii* | 100% | Muranga (adult) |
| BD2 | MK968292 | Proteobacteria | Enterobacteriaceae | *Enterobacter tabaci* | 100% | Kitui, Makueni (adult) |
| BD3* | MK968293 | Proteobacteria | Enterobacteriaceae | *Enterobacter cloacae* | 100% | *icipe*, Makueni, Kitui (adult) |
| BD4* | MK968294 | Proteobacteria | Enterobacteriaceae | *Klebsiella oxytoca* | 100% | Makueni, *icipe* (adult) |
| BD5 | MK968295 | Firmicutes | Streptococcaceae | *Lactococcus lactis* | 100% | Embu (adult) |
| BD6 | MK968296 | Firmicutes | Streptococcaceae | *Lactococcus lactis* | 99.80% | Embu (adult) |
| BD7 | MK968297 | Firmicutes | Streptococcaceae | *Lactococcus lactis* | 99.80% | Embu (adult) |
| BD8* | MK968298 | Firmicutes | Streptococcaceae | *Lactococcus lactis* | 100% | Nguruman (adult) |
| BD9 | MK968299 | Firmicutes | Streptococcaceae | *Lactococcus lactis* | 100% | Embu (adult) |
| BD10* | MK968300 | Proteobacteria | Enterobacteriaceae | *Providencia alcalifaciens* | 100% | Muranga (larvae) |
| BD11 | MK968301 | Proteobacteria | Enterobacteriaceae | *Providencia rettgeri* | 100% | Muranga (larvae) |
| BD12 | MK968302 | Proteobacteria | Enterobacteriaceae | *Enterobacter asburiae* | 100% | Muranga, Nguruman, Makueni, Kitui (adult) |
