# Supplementary material for: Influence of inoculated gut bacteria on the development of Bactrocera dorsalis and on its susceptibility to the entomopathogenic fungus, Metarhizium anisopliae
Source: BMC Microbiol. 2020 Oct 21;20:321. doi: 10.1186/s12866-020-02015-y (PMC7579797; doi:10.1186/s12866-020-02015-y)
Supplement: Supplementary file 6 — Additional file 6 Supplementary Table 2: Pairwise comparison of survival rates between bacterial-inoculated B. dorsalis lines, post exposure to entomopathogenic fungus. [file 12866_2020_2015_MOESM6_ESM.docx]

Supplementary Table 2: Pairwise comparison of survival rates between bacterial-inoculated *B. dorsalis* lines, post exposure to entomopathogenic fungus.

| ***B. dorsalis* lines** | χ **^2^** | **df** | ***P* value** |
| --- | --- | --- | --- |
| Ut-control and *C. freundii* | 0.009 | 1 | 0.92 |
| Ut-control and *E. cloacae* | 0.03 | 1 | 0.95 |
| Ut-control and *K. oxytoca* | 0.14 | 1 | 0.70 |
| Ut-control and *L. lactis* | 3.40 | 1 | 0.07 |
| Axenic and *C. freundii* | 0.37 | 1 | 0.54 |
| Axenic and *E. cloacae* | 0.38 | 1 | 0.54 |
| Axenic and *K. oxytoca* | 0.95 | 1 | 0.33 |
| *C. freundii* and *E. cloacae* | 0.003 | 1 | 0.95 |
| *C. freundii* and *K. oxytoca* | 0.20 | 1 | 0.65 |
| *C. freundii* and *P. alcalifaciens* | 3.40 | 1 | 0.07 |
| *E. cloacae* and *K. oxytoca* | 0.21 | 1 | 0.65 |
| *E. cloacae* and *L. lactis* | 3.75 | 1 | 0.05 |
| *E. cloacae* and *P. alcalifaciens* | 3.06 | 1 | 0.08 |
| *K. oxytoca* and *L. lactis* | 2.08 | 1 | 0.15 |
|  |  |  |  |
